# An Efficient Hybrid Feature Selection Method Using the Artificial Immune Algorithm for High-Dimensional Data

**DOI:** 10.1155/2022/1452301

**Published:** 2022-10-13

**Authors:** Yongbin Zhu, Tao Li, Wenshan Li

**Affiliations:** ^1^College of Cybersecurity, Sichuan University, Chengdu 610065, China; ^2^Engineering College, Honghe University, Mengzi 661199, China

## Abstract

Feature selection provides the optimal subset of features for data mining models. However, current feature selection methods for high-dimensional data also require a better balance between feature subset quality and computational cost. In this paper, an efficient hybrid feature selection method (HFIA) based on artificial immune algorithm optimization is proposed to solve the feature selection problem of high-dimensional data. The algorithm combines filter algorithms and improves clone selection algorithms to explore the feature space of high-dimensional data. According to the target requirements of feature selection, combined with biological research results, this method introduces the lethal mutation mechanism and the Cauchy operator to improve the search performance of the algorithm. Moreover, the adaptive adjustment factor is introduced in the mutation and update phases of the algorithm. The effective combination of these mechanisms enables the algorithm to obtain a better search ability and lower computational costs. Experimental comparisons with 19 state-of-the-art feature selection methods are conducted on 25 high-dimensional benchmark datasets. The results show that the feature reduction rate for all datasets is above 99%, and the performance improvement for the classifier is between 5% and 48.33%. Compared with the five classical filtering feature selection methods, the computational cost of HFIA is lower than the two of them, and it is far better than these five algorithms in terms of the feature reduction rate and classification accuracy improvement. Compared with the 14 hybrid feature selection methods reported in the latest literature, the average winning rates in terms of classification accuracy, feature reduction rate, and computational cost are 85.83%, 88.33%, and 96.67%, respectively.

## 1. Introduction

With the continuous in-depth understanding of the research object and the development of data acquisition technology, high-dimensional data has become more and more common. In theory, the more information obtained, the more conducive it is to obtain a more accurate judgment of the object. However, the actual situation may be far from it, because there are often many redundant data irrelevant to the research objectives in these data. They play the role of noise in the pattern recognition model and greatly increase the computational cost of the model. Moreover, in some cases, they will guide the learning process in the model to the generation direction of a weak model, resulting in wrong results. In addition, too many features will not only increase the computational cost in the stage of model training and feature analysis but also lead to the complexity of the model. This can easily lead to the problems of “dimension disaster” and “overfitting” [1]. Reducing the dimension of data is an effective way to solve the classification problem of high-dimensional small sample data [2]. Feature selection technology is widely used to deal with such problems. The goal of feature selection is to select as few features as possible to effectively describe the whole feature space [3]. It can greatly reduce the time of the model in the training stage while maintaining or even improving the classification accuracy.

With the deepening of research and the expansion of application fields, feature selection has been considered to be an important data preprocessing step in the fields of pattern recognition and machine learning [4]. A variety of feature selection methods have been proposed for different application fields to improve the recognition performance of the model. From a broad point of view, these methods may generally be divided into filter and wrapper methods. In practical application, they have their own advantages and disadvantages. The filter method uses feature correlation criteria to select feature subsets with lower computational costs. The wrapper method uses the classification algorithm to evaluate the quality of the selected features, so as to obtain a higher quality feature subset. In recent years, feature selection methods based on metaheuristics have been the focus of scholars because of their good global search ability [5]. These algorithms include simulated annealing algorithm (SA), genetic algorithm (GA), particle swarm optimization (PSO), and artificial immune algorithm (IA). They all have good global searchability and do not need to provide domain knowledge or prior assumptions about the search space. Moreover, they can effectively deal with complex problems that are difficult to be solved by traditional optimization algorithms without being limited by the nature of the problem. Many literature studies show that feature selection methods based on metaheuristics have excellent performance in solving common feature selection problems. However, with the expansion of search space, especially when the number of features reaches thousands, its calculation cost will increase exponentially [6].

To sum up, the feature selection of high-dimensional data has the following problems. First, the feature subsets obtained by the filter method have low accuracy, which requires artificial analysis of different datasets and selection of specific filter threshold values for them. Second, the metaheuristic-based wrapper method suffers from the problem of high computational cost. In order to solve the above problems, combined with the advantages of the filtering and wrapper method, this paper proposes an efficient hybrid feature selection method based on artificial immune algorithm optimization, namely, HFIA. The algorithm combines the Fisher filtering algorithm and an improved clonal selection algorithm to explore the search space of the optimal feature subset. The Fisher algorithm is an effective filtering feature selection method. It identifies the importance of features by calculating the mean and variance of the distance between and within classes. The artificial immune algorithm is an efficient optimization algorithm that simulates the function of the natural immune system. It is an intelligent algorithm inspired by the principle, function, and model of biological immunity. Based on the traditional evolutionary algorithm, it introduces the mechanism of affinity maturity, cloning, and memory. It has the characteristics of fast convergence speed and strong global optimization ability. It is widely used to solve problems related to optimization and pattern recognition.

According to the target requirements of feature selection, this paper greatly improves the clonal selection algorithm. These improvements include population initialization, mutation strategy, and population update mechanism of antibodies. Combined with the research results of biology, this paper introduces the lethal mutation mechanism and the Cauchy operator to improve the search performance of the algorithm. And different adaptive adjustment factors are introduced in the mutation and update phases of the algorithm. They are used to improve the search speed of the algorithm and enhance the diversity of the population, respectively. The effective combination of these strategies enables the algorithm to obtain better searchability and lower computational costs. The evaluation results on 25 high-dimensional datasets with features ranging from 2000 to 22283 demonstrate the effectiveness of this method. It is compared with 5 classical feature selection methods and 14 hybrid feature selection methods for high-dimensional data reported in the latest literature. The results show that the computational cost of this algorithm is comparable to classical feature selection methods known for their speed. Moreover, it achieves better average classification accuracy than hybrid feature selection methods reported in the latest literature with the smallest number of optimal feature subsets. The comparative experimental results fully demonstrate the progressiveness of the algorithm.

The rest of this paper is organized as follows. Firstly, the feature selection methods and their related domain knowledge are summarized in Section 2. Secondly, the implementation details of the proposed method are described in Section 3. Then, Section 4 provides a detailed description of the experimental datasets, the evaluation metrics, and the algorithm parameter settings. The experimental results are analyzed and discussed in Section 5. Finally, Section 6 gives the conclusion.

## 2. Preliminaries and Related Work

In this section, feature selection techniques based on metaheuristic algorithms are reviewed first. And the problems of this kind of method in feature selection in high-dimensional data space are proposed. Secondly, the related work of current hybrid feature selection methods is summarized. Finally, the principle of the immune clonal selection algorithm is introduced.

### 2.1. Feature Selection Based on Metaheuristic Algorithms

Feature selection can filter out the best feature subset that can represent the whole dataset by removing irrelevant or unnecessary features. It is considered to be one of the most critical and challenging problems in machine learning. It is widely used to solve the problem of dimension reduction of datasets in different fields, such as the best gene screening in biomedicine [7], the hot topic recognition in text mining [8], and the best visual content pixel and color selection in image analysis [9]. These algorithms are mainly divided into the filter and wrapper method. The filter method focuses on the internal relationship of data. They are usually not directly related to learning algorithms or classification algorithms. They use different correlation criteria of features to select the optimal feature subset. Therefore, most of them have the advantage of low computing costs. The wrapper method focuses on the interaction results between different feature combinations and classifiers. Compared with the former, these methods need to pay higher computational costs, but they can provide more accurate results. The generation of the optimal subset of the wrapper method is based on a specific search strategy. These search technologies are mainly divided into three categories. They are the exponential selection strategy, sequential selection strategy, and random selection strategy [10]. In recent years, the metaheuristic algorithms have been widely used in the problem of selecting optimal subsets of feature spaces [5].

They are derived from the heuristic algorithm and are also the product of the combination of the random algorithm and the local search algorithm. They have a good global search ability, and there is no need to provide domain knowledge or advance assumptions about the search space. A metaheuristic algorithm is also called an intelligent optimization algorithm. They are based on the mechanism of computational intelligence to solve some complex optimization problems. The solution obtained by this kind of algorithm is called the optimal solution or satisfactory solution. They have the advantages of simple concepts and easy implementation. In addition, they also have the features of flexibility and intuition and can be modified according to the specific problems that are to be solved. Moreover, they also have the significant advantage of preventing the algorithm from falling into the local optimal solution due to premature convergence, so they can effectively explore the whole problem space. Because of these advantages, the metaheuristic algorithm has attracted extensive attention from researchers. Metaheuristic-based optimization algorithms have also been successfully applied to various engineering and scientific research optimization problems, such as job scheduling, transportation management, vehicle path planning and facility location in industrial engineering, bridge structure and architectural design in civil engineering, radar design and networking in communication engineering, classification, prediction, clustering, and system modeling in data mining.

Feature selection methods are usually implemented by searching the solution space with the goal of maximizing the correlation with the target class and minimizing the redundancy of the selected features [11]. Although this goal can be achieved through the simplest exhaustive search strategy. However, its calculation cost is unacceptable. This is even more unrealistic for large-scale datasets. Since it is too expensive to evaluate all possible feature subsets, a method that is acceptable in terms of computational complexity needs to be used to find suitable feature subsets [12]. The metaheuristic algorithm provides an effective way to solve this kind of problem. It can find a satisfactory near-ideal solution in an acceptable time, although this is not the only optimal solution [13].

Most metaheuristics start by generating several random initial solutions and then evaluating the resulting set of solutions using a fitness function [14]. The approximate optimal solution is searched by continuous loop iteration until one of the termination conditions is satisfied. In addition, people always want to get better results from machine learning models. The strategy of adding different optimization objectives to the fitness function of the feature selection problem emerges as the times require. Using a multi-objective optimization strategy to model the feature selection problem, a set of nondominated feature subsets can be obtained. This solution can meet various requirements in practical applications when the number of features is not too large. However, as the data dimension continues to expand, especially when the number of features reaches thousands, the computational cost will increase exponentially. Therefore, it is urgent to find a more efficient solution to the feature selection problem in high-dimensional data space.

### 2.2. Hybrid Feature Selection Approaches

Because of the particularity of high-dimensional data space, it is difficult to provide a satisfactory solution whether it is a filter method, a wrapper method, or any other single feature selection method. In recent years, hybrid algorithms have received extensive attention in solving optimization problems. Hybrid algorithms are those that combine different algorithms and develop a new or improved algorithm to solve more complex optimization problems.

In the problem of feature selection, a variety of hybrid algorithms have been used to solve the problem of selecting the optimal feature subset of high-dimensional data. The hybrid feature selection method combines the advantages of different methods. The hybrid scheme of multiple algorithms greatly increases the probability of finding the optimal solution efficiently and quickly. Moreover, some hybrid algorithms combine the best characteristics of different algorithms to develop new algorithms. Therefore, the hybrid algorithm can greatly reduce the search space of the optimal feature subset. In this case, the hybrid algorithm based on a metaheuristic algorithm reduces the possibility of falling into the local optimal solution. This is because when a large number of noisy data are removed, they can better avoid premature convergence and explore the whole data space more effectively. In most cases, the hybrid algorithm can obtain the optimal solution with better quality. Moreover, they can make a better trade-off between search quality and development quality of the algorithm. Therefore, compared with the single feature selection method, the hybrid method has a better application value.

So far, a variety of hybrid feature selection methods have been proposed. Combining global search and local search methods, Liu et al. [15] proposed a hybrid feature selection method based on a genetic algorithm and embedded regularization. Lu et al. [16] proposed a hybrid feature selection algorithm that combines mutual information maximization and an adaptive genetic algorithm. The algorithm first removes a large number of redundant features by maximizing mutual information and then searches for the optimal feature subset by an adaptive genetic algorithm. Ma et al. [17] proposed a two-stage hybrid ant colony algorithm for high-dimensional feature selection. It uses the interval strategy to determine the size of the optimal feature subset searched in the additional stage. This method helps to reduce the complexity of the algorithm and avoid the algorithm falling into local optimization. Huang et al. [18] designed a two-stage hybrid feature selection algorithm. This method combines the binary state transition algorithm and the ReliefF algorithm and shows a good performance on low-dimensional data with the help of new operators. In addition, Yan et al. [19] proposed a hybrid feature selection algorithm combining simulated annealing and an improved coral reef optimization algorithm. The algorithm is used to solve the feature selection problem of high-dimensional biomedical datasets. Hussain et al. [20] proposed a hybrid optimization method integrating the sine-cosine algorithm into Harris hawks. The method is used to solve the problems of numerical optimization and feature selection. Song et al. [21] proposed a hybrid feature selection based on correlation-guided clustering and particle swarm optimization, which is used to solve the feature selection problem of high-dimensional data. Xiao et al. [22] proposed a hybrid feature selection method that fuses multiple algorithms. The method first filters the feature space by combining the k-means clustering algorithm and the signal-to-noise ratio ranking method, and then combines the cellular learning automaton with the ant colony optimization as a wrapper method to apply to the reduced dataset. Chen et al. [23] proposed a hybrid feature selection method based on evolutionary multitasking. The method first uses the ReliefF algorithm to calculate feature weights and then searches for the optimal feature subset through PSO. Wan et al. [24] proposed a hybrid feature selection method that combines neighborhood rough sets and conditional mutual information. The method first uses various neighborhood information to measure and redefine the correlation of features and then obtains the optimal feature subset through the interactive feature selection algorithm (NCMI_IFS) based on neighborhood conditional mutual information. Song et al. [25] proposed a hybrid feature selection algorithm based on surrogate sample-assisted particle swarm optimization (SS-PSO). The method first uses a cooperative feature clustering mechanism to divide the feature space and then uses PSO to search different feature spaces to obtain the optimal feature subset.

These hybrid methods can overcome the shortcomings of traditional methods in solving the feature selection of high-dimensional data to a certain extent. Compared with the classical filter feature selection method and other methods based on an intelligent algorithm, they can reduce feature redundancy and computational cost to a greater extent. Moreover, they have a better performance in improving the classification performance of the classifier. However, they still have the problem that the classifier performance is not ideal due to too much noise in the selected feature subset to varying degrees. In addition, most algorithms also have the problem of high computational cost due to the complexity of the algorithm itself or insufficient optimization.

### 2.3. Artificial Immune Optimization Algorithm

The artificial immune algorithm is an intelligent computing method designed to solve complex optimization problems. It is also a metaheuristic algorithm that simulates the operation mechanism of the biological immune system. Since Burnet [26] fully elaborated the principle of clone selection in 1959, the algorithm has been generally recognized by the immunology community. This theory states that B cells with a high affinity for antigens in organisms are retained by the immune system and have the characteristics of clonal proliferation, and these proliferating cells will differentiate into two types of cells with different functions. Some are memory cells that function as antigen markers. Others are plasma cells that destroy antigens, known as antibodies. The theory of clonal selection is used to explain the characteristics of immune responses to antigenic stimulation. The core idea is to select only those cells that can recognize antigens for cloning and proliferation. It describes the properties of the acquired immunity of the biological immune system. The clonal selection mechanism corresponds to the process of affinity maturation of immune cells. That is, under the action of this mechanism, immune cells with lower affinity to antigens undergo a process of “maturing” by gradually increasing their affinity after undergoing clonal proliferation and mutation. During this process, mutations in cloned individuals are inversely proportional to antigen affinity. The production of antibodies is the learning process of the immune system.

Based on clonal selection theory, de Castro and Von Zuben proposed a famous clonal selection algorithm (CLONALG, also known as CSA) in 2000 [27]. It is pointed out in literature that the algorithm is mainly composed of population initialization, clone selection, clone proliferation, hypermutation, and population renewal. Among the important features of the clonal selection algorithm, the hypermutation is an important part. It is the basic guarantee to realize the diversity of algorithms, but the choice is the premise.

The basic clonal selection algorithm consists of the following steps [28]. Each time these steps are performed, a new generation of immune cells will be generated.

① Antibody initialization: generate a set P of candidate solutions.
② Affinity evaluation: calculate the affinity of each antibody in the antibody pool.
③ Selection and cloning: select *n* antibodies with the highest affinity, and clone these *n* antibodies in proportion to their affinity with the antigen to form a clone group C.
④ Hypermutation: the clone population is proposed to hypermutation operation, and a mature antibody population D is generated.
⑤ Population update: D is reselected to form a memory cell set M. *d* new antibodies are generated to replace the lower affinity antibodies in P.
⑥ Repeat steps 2–5 until the termination condition is met.

In recent ten years, the clonal selection algorithm has attracted the attention of many researchers because of its good global optimization ability and convergence performance. Accordingly, the clonal selection algorithm has evolved into many variants and applied to different research fields. Shang et al. [29] improved the basic CSA in terms of population initialization, clonal selection method, and population update, so as to obtain better convergence when solving multi-objective optimization problems. Dai et al. [30] proposed a clonal selection algorithm based on the bidirectional quantum crossover. In this method, the bidirectional quantum crossover mechanism in quantum jump theory is used to replace the hypermutation operation to realize the information exchange between antibodies. And to improve the search performance of the CSA algorithm. Xu et al. [31] adopted the degradation identification (DR) method to evaluate the suboptimal solution to be eliminated and thus improved the computational efficiency of the CSA algorithm in dealing with complex engineering multimodal optimization problems. Yan et al. [32] improved the clonal selection algorithm and successfully applied it to solve the nonlinear optimization problem in AVO elastic parameter inversion. Luo et al. [33] improved the basic clone selection algorithm to solve the global optimization problem in dynamic multimodal optimization.

## 3. Proposed HFIA

In this paper, a hybrid feature selection method (HFIA) combining the filter feature selection method with a multi-objective artificial immune algorithm is proposed. This method effectively combines the advantages of the Fisher filtering algorithm and the improved clonal selection algorithm. According to the target requirements of feature selection in high-dimensional data, this method greatly improves the initialization and mutation strategy of antibody population of the clonal selection algorithm. In this section, the implementation details of the HFIA algorithm and several improvements to the clonal selection algorithm will be described in detail. These descriptions include the following aspects: antibody coding mode, affinity evaluation operator, population initialization mode, mutation strategy, and update mechanism of mature antibodies. Finally, the pseudo-code of the algorithm is given at the end of this section, and the symbols used in the algorithm are also explained.

### 3.1. Solution Encoding

For feature selection methods based on metaheuristic algorithms, binary coding strategy is mostly used to represent feature space. This is because binary vectors can not only easily represent subsets of features but also simplify the operation of the algorithm. Therefore, for feature selection problems, binary encoding is usually adopted to represent the individuals in the solution. In this paper, we also adopt the binary coding strategy. Moreover, this can also make better use of the advantages of the algorithm itself.

In this paper, the encoding is a binary vector of length *n*. Each bit corresponds to a feature, and *n* is the size of the feature space. The code uses a value of “0” or “1” to characterize whether the feature at that location is included in the feature subset. A value of “1” indicates that the feature at this location is selected, otherwise it is not selected.

### 3.2. Evaluating the Fitness

In this paper, a multi-objective optimization strategy is used to model the feature selection problem. The purpose is to obtain a subset of features with a smaller number of features while striving to achieve higher classification accuracy. Therefore, for the feature selection problem, we have two optimization objectives, which are classification accuracy and the number of feature subsets. According to the multi-objective optimization decision-making model introduced above, this paper constructs the following formula (1) as the fitness function. The construction method of the fitness function is also widely used in other literature [34–37] to evaluate the quality of feature subsets.

(1) $$\begin{array}{c} fitness=\omega \times E_{r}+\left(1-\omega \right)\times \left(\frac{p}{q}\right)\text{.} \end{array}$$

Among them, *ω* ∊ (0, 1) is a given real number. In most literature, *ω* is usually set to 0.9 [38]. *E*_*r*_ is the classification error rate. It is obtained by evaluating this subset of features by an evaluator (usually a classifier). *q* is the total number of features in the dataset, while *p* is the number of selected features in the feature subset. In this paper, we use K-nearest neighbor (KNN) as a classification estimator for feature subsets, where *k* = 5.

### 3.3. Initial Population Generation

In the basic clonal selection algorithm, the initial population is generated by random distribution. In fact, many biological phenomena appear in the form of probability distribution of continuous random variables. In addition, the probability distribution of many random variables takes the normal distribution as its limit distribution under certain conditions. The normal distribution is also known as the Gaussian distribution. The Gaussian distribution is a very important probability distribution in many fields such as mathematics, physics, and engineering. In evolutionary computing, Gaussian distribution is often used in the population mutation link of evolutionary algorithms. Extensive literature studies have shown that large mutational situations in populations are less likely when a Gaussian distribution is used. This may cause the problem of insufficient algorithm diversity. This increases the risk of the search algorithm falling into a local optimum and reduces the convergence speed of the algorithm. Cauchy distribution is another continuous probability distribution function. Compared with Gaussian distribution, its attenuation speed is slower and allows a larger mutation step. This greatly increases the possibility of the algorithm jumping out of the local optimum. Moreover, it has been reported that even in terms of the diversity of methods, the Cauchy distribution is better than the Gaussian distribution in the search process of the evolutionary algorithm [39–41]. The probability density function of the one-dimensional Cauchy distribution is shown in the following formula:

(2) $$\begin{array}{c} f\left(x;x_{0},\gamma \right)=\frac{1}{\pi \gamma \left[1+{\left(x-x_{0}/\gamma \right)}^{2}\right]}=\frac{1}{\pi }\left[\frac{\gamma }{{\left(x-x_{0}\right)}^{2}+\gamma^{2}}\right]\text{.} \end{array}$$

The Cauchy distribution has two parameters, *x*_0_ and *γ*. *x*_0_ is the position parameter and *γ* is the scale parameter. They determine the shape of the Cauchy distribution. If the value of *γ* is larger, the peak height of the probability density function will be smaller and the width will be larger. Conversely, if the value of *γ* is small, the peak height of the probability density function will be higher, and the peak width will be smaller. When *γ* = 1 and *x*_0_ = 0, it is called the standard Cauchy distribution. Its probability density function is shown in the following formula:

(3) $$\begin{array}{c} f\left(x;0,1\right)=\frac{1}{\pi \left(1+x^{2}\right)}\text{.} \end{array}$$

Its corresponding cumulative distribution function is shown in the following formula:

(4) $$\begin{array}{c} F\left(x;x_{0},\gamma \right)=\frac{1}{\pi }\arctan\left(\frac{x-x_{0}}{\gamma }\right)+0.5\text{.} \end{array}$$

**Figure 1:**
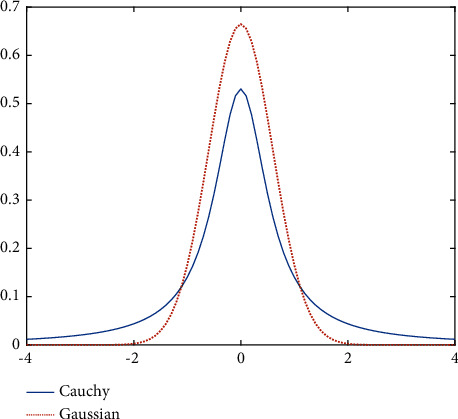
Comparison of probability density functions between Cauchy distribution and Gaussian distribution.

When the parameters are the same, the probability density functions of the Cauchy and Gaussian distributions are shown in Figure 1. The following conclusions can be drawn intuitively from the figure. Compared with the Gaussian distribution, the Cauchy distribution has a slower decay rate and a larger range of values.

Therefore, in order to obtain the optimal feature subset more quickly, the Cauchy distribution will be applied to the initialization, mutation, and update stages of the population in this paper. At the same time, this is also to reduce the computational cost of the algorithm. The initial population space is generated using the standard Cauchy distribution, which is then transformed into a feature code for antibodies. The standard Cauchy distribution function is shown in formula (3). The outline of the population initialization algorithm is given in Algorithm 1. Firstly, the algorithm generates the real initial population space through the standard Cauchy distribution function. Then, according to the threshold value *η*, the real number bits in the initial population space are converted into binary bits that can represent the feature code. In this paper, the value of *η* is −0.2. That is, when *η* > −0.2, the locus of the antibody is assigned with a value of 1, otherwise, it is 0.

### 3.4. Mutation and Update Strategy

The clonal selection algorithm introduces the mutation theory of organism cells to promote the proliferation and evolution of individuals in the population. The mutation mechanism plays an important role in the operation steps of the clone selection algorithm. It gives the algorithm the capability of local random search. At the same time, it also has the function of maintaining the diversity of the population and preventing the phenomenon of premature convergence of the algorithm. Mutations of individuals in a population are carried out at randomly selected loci. Its fundamental purpose is to make the population more diverse. Hypermutation is an important mechanism for the biological immune system to recognize external invasion. It obtains a higher affinity for the antigen through the mutation mechanism of the antibody gene. Due to the semi-blindness of the clone selection algorithm in the search problem, scholars have proposed various mutation strategies to improve the algorithm. These mutation strategies usually have differences for different problem domains.

The fundamental purpose of feature selection is to find a better subset of features to represent the entire feature space, that is, to find a subset with less feature redundancy and higher classification accuracy. Based on this, this paper is inspired by the phenomenon of lethal mutation in gene mutation theory and performs lethal mutation operations on elite antibodies in the population. In this way, the algorithm is accelerated to search in the direction of a smaller number of feature subsets. From a biological point of view, although lethal mutations are detrimental to lethal individuals, they are beneficial for maintaining the heterozygous state of the population. The experimental results show that it can make the algorithm obtain better one-way search ability in solving the feature selection problem of high-dimensional data. Therefore, a feature subset with less feature redundancy can be obtained while ensuring classification accuracy. Moreover, it can also reduce the computational cost of the algorithm to a greater extent.

Furthermore, to steer the mutation process in the direction required by the problem domain, an adaptive linear acceleration factor *δ* ∈ (-0.5, 0.5] was added to the mutation process of the elite antibodies. Its effect is to accelerate the decay rate of the genes of the elite antibodies. It works on the condition that the affinity of the locally optimal antibody continues to increase. That is, under the condition of ensuring the classification performance of the optimal feature subset, it is accelerated to search in the direction of a smaller number of features. The calculation formula of the acceleration factor is shown in the following formula:

(5) $$\begin{array}{c} \delta =0.5-\frac{t}{T_{\max}}\text{.} \end{array}$$

Among them, *t* is the current number of iterations, and *T*_max_ is the total number of iterations.

**Figure 2:**
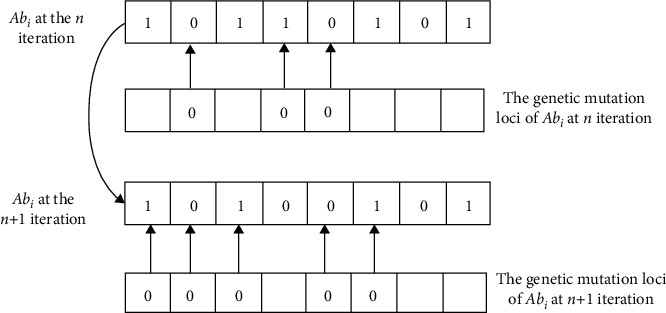
The schematic diagram of conditional lethal mutation of an antibody.

As the number of iterations increases, the value of *δ* will gradually tend to −0.5 from 0.5. In each iteration process, it is first necessary to determine the mutation loci of each antibody in mutation set *C*. The mutation loci are jointly determined by the generated Cauchy random number sequence and the transformation threshold value *δ*. Therefore, the threshold *δ* has an important effect on the position and quantity of the mutated loci of an antibody. It can be seen from the schematic diagram of the probability density function of the Cauchy distribution in Figure 1. When the value of the conversion threshold is smaller, there will be more “1” loci in an antibody, that is, the more loci involved in mutation, and vice versa. Therefore, when the conditional lethal mutation is used, the algorithm will speed up the search in the direction of fewer features. When the fitness value change of the local optimal antibody meets the mutation conditions, Figure 2 describes the genetic changes when an antibody performs lethal mutation operation.

Algorithm 2 lists the main steps to perform a lethal mutation operation on the elite antibodies selected from the population in each iteration.

In the classical clonal selection algorithm, the update of the population is carried out on the premise of maintaining the population number unchanged. In the HFIA algorithm, since the using of a lethal mutation strategy, the number of genes in the antibody decays rapidly during the mutation process. The purpose is to guide the algorithm to search in the direction of fewer features, so as to obtain high-quality feature subsets with fewer features. Compared with other intelligent algorithms based on metaheuristics, the search of the algorithm is not completely random. The advantage of this strategy is that the algorithm's search is better guided. On the other hand, this also helps to reduce the complexity and the computational cost of the algorithm itself. But correspondingly, it is also easier to cause the algorithm to fall into a locally optimal solution. In order to eliminate the risk of falling into a local optimum due to excessively rapid fitness decay, it is necessary to enhance the diversity of the population during the iterative process.

Therefore, two strategies are adopted to compensate. On the one hand, the size of the population is expanded when the population is updated. To this end, the strategy of incremental update is adopted. That is, the number of updates for HFIA is N, while the number of updates in the classical algorithm is *d* (*d* < *N*). This does not mean that the population will continue to increase during iterations. Selection is adopted to keep the overall size of the population constant. On the other hand, a linear incremental regulator *θ* is added in the population update phase. Its purpose is to dynamically adjust the number of antibody genes that is newly added to the population according to iterative changes. That is, the mutation probability of individuals in the population is enhanced, so as to achieve the purpose of improving the diversity of the population. The calculation formula of the adjustment factor is shown in the following formula:

(6) $$\begin{array}{c} \theta =\frac{t}{T_{\max}}\text{.} \end{array}$$

Among them, *t* is the current number of iterations, and *T*_max_ is the total number of iterations. For its specific implementation and application, please refer to the algorithm framework code part in next section.

### 3.5. The Proposed Algorithm Framework and Notation

**Figure 3:**
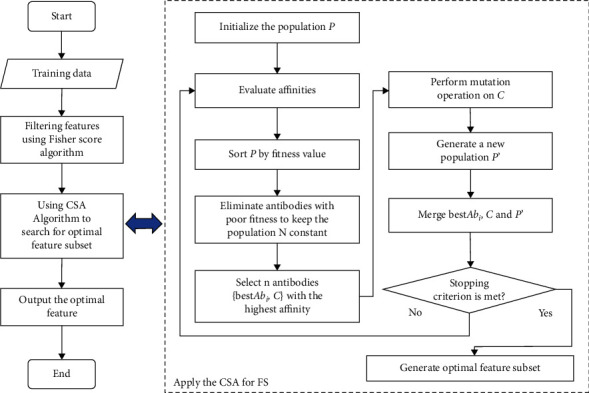
Framework of HFIA.

In theory, the higher the dimension of the data, the more detailed the description of things. This plays an important role in some fields of research. But for classification problems, too much redundant feature data will cause a serious decline in the performance of the classifier and even lead to the problem of dimensional disaster. For the feature selection problem in high-dimensional data space, the main idea is to use a hybrid feature selection method. But how to combine different algorithms more effectively is worthy of further study by scholars. Through many experiments, this paper finds a more efficient hybrid feature selection method than the current literature reports to solve the feature selection problem of high-dimensional data. The method solves the problem of selecting optimal feature subsets for high-dimensional data through a two-stage screening operation. The HFIA algorithm framework is shown in Figure 3.

The algorithm evaluates and ranks all features in the data space by the Fisher scoring function in the first stage. The Fisher score algorithm calculates the mean and variance of the distances between different categories of features and within the same category. It identifies the importance of features through the calculated mean and variance. It is an effective filtering feature selection method and has the advantage of fast calculation speed. The calculation method will be briefly introduced in [42].

Given a set of labelled data samples, {*Ab*_*i*_, *Ab*_*j*_}, *Ab*_*j*_ ∈ {1,…, *c*}, *i*=1,…*n* , where *c* is the number of categories, and *n*_*k*_ represents the number of data samples in the *k*th category. *u*_*k*_^*i*^ represents the mean of all data samples on the *i*th feature. *μ* and *σ* are the mean and variance of the category *k* corresponding to the *i*th feature, respectively. The Fisher score of the *i*th feature can be calculated by the following formula:

(7) $$\begin{array}{c} F_{k}=\frac{\sum_{k=1}^{c}n_{k}{\left(u_{k}^{i}-u^{i}\right)}^{2}}{\sum_{k=1}^{c}n_{k}{\left(\sigma_{k}^{i}\right)}^{2}}\text{,} \end{array}$$

where ∑_*k*=1_^*c*^*n*_*k*_(*u*_*k*_^*i*^ − *u*^*i*^)^2^ is the variance of the *i*th feature between different categories, and ∑_*k*=1_^*c*^*n*_*k*_(*σ*_*k*_^*i*^)^2^ is the variance of the *k*th feature within the same category.

**Figure 4:**
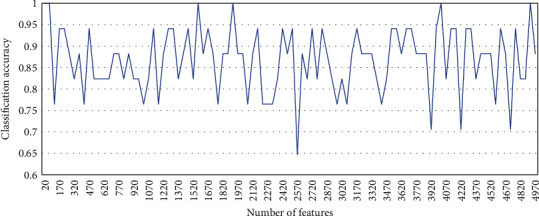
Relationship between the Fisher score screening threshold and classification accuracy of GLI-85.

After the classification importance scores of the features are obtained, the features can be filtered to obtain a reduced subset of candidate data. Compared with the full feature set, the feature subset obtained through the first stage has been greatly reduced in the number of features. Theoretically, for any filtering-type feature selection method, as long as an optimal threshold value is selected, the desired feature subset can be obtained. Although the selection of this optimal threshold value can be achieved by a simple exhaustive method, there is no guarantee that the feature combined with a high score is the one with the best quality. Because the feature score obtained by any univariate evaluation rule does not guarantee that the combination with a higher score is the optimal feature subset. In addition, experimental verification was performed to address this issue. On different datasets, the feature subsets are screened and classified by increasing the threshold. The experimental results show that the classification accuracy of the features scored and sorted by the Fisher score always oscillates within a certain range after being screened by different thresholds. Moreover, the peak value of its oscillation does not have a linear proportional relationship with the selected threshold value. The following Figure 4 is the relationship between the increase of the threshold value of the Fisher score of GLI-85 and the classification accuracy. The experimental results on other datasets are similar to this figure. It will not be repeated here.

Therefore, this paper adopts the artificial immune algorithm with a good global search performance to perform a secondary search on the feature subset after the initial screening. A hybrid feature selection method based on the Fisher filtering method combined with the wrapper method optimized by the artificial immune algorithm is constructed. After experimental analysis, considering both the quality of the optimal feature subset and the computational cost of the algorithm itself, this paper chooses the filter threshold value of the Fisher score to be 200. The structural framework and main steps of the HFIA algorithm are shown in the following algorithm 3.

In addition, Table 1 describes the important identifiers used in the algorithm.

## 4. Experiment Methodology

In this section, datasets used in the experiment are first introduced, then the performance evaluation criteria of the classification test are explained, and finally, the parameters setting of the HFIA algorithm in the experiment are described.

### 4.1. Datasets

In the experiments in this paper, a total of 25 real datasets are used to verify the performance of the proposed feature selection algorithm. These datasets cover varying numbers of features from 2000 to 22283. They are datasets from UCI Repository [43], feature selection datasets from Arizona State University [44], microarray datasets [45], and gene expression datasets [23], respectively. The UCI dataset is used in the evaluation of feature selection algorithms in many pieces of literature. In addition, the ASU feature selection dataset, microarray dataset, and gene expression dataset are specially selected to examine the performance of the algorithm on high-dimensional datasets.  Table 2 shows the details of these datasets.

### 4.2. Performance Evaluation Criteria

In this paper, the cross-validation [46] is used to evaluate the accuracy of the classification algorithm. It is a commonly used validation technique and is widely used to evaluate the performance of machine learning models. The average classification accuracy of KNN is used to evaluate the quality of the selected optimal feature subset in this paper. The classification accuracy, the number of features of the optimal feature subset, and the average and deviation of the computational cost obtained from the experimental results are all statistical results after the algorithm runs 20 times independently on each dataset. And based on these statistical results, the performance of the algorithm is evaluated. For other parameters in the comparative experiment, the setting values described in the corresponding literature are used.

### 4.3. Parameter Settings

All experiments are performed on a PC with an Intel Core i5 and 8 GB of RAM. Also, all algorithms are performed on different datasets using the same settings. In all experiments, the parameter configuration of the HFIA algorithm is as follows. The maximum number of consecutive iterations of the algorithm is *T* = 50, the population size *N* = 10, the select rate *c*_*r*_ = 0.5, and the initial transformation threshold value of Cauchy random numbers *η* = −0.2. The parameter *w* of the fitness function is set to 0.99. According to the experimental analysis of the Fisher algorithm in Section 3.5, this paper sets its filter threshold value to be 200.

## 5. Experiments and Discussion

In this section, the proposed feature selection method HFIA is comprehensively evaluated and analyzed through experiments. Firstly, the performance of HFIA on all 25 datasets involved in the experiment is analyzed. The experimental results are compared with the results using full features. These comparisons include the reduction degree of redundant features and the improvement of the classifier performance. Secondly, the HFIA algorithm is compared with various feature selection methods reported in other literature. These feature selection methods include several classical univariate filtering feature selection algorithms and a variety of hybrid feature selection methods reported in the latest literature. These analyses and comparisons include the following three aspects. They are the classification quality and the number of features of the optimal feature subset obtained, as well as the computational cost of the algorithm. In all tabular data, the best results of each standard are identified in bold.

### 5.1. Performance Evaluation

**Figure 5:**
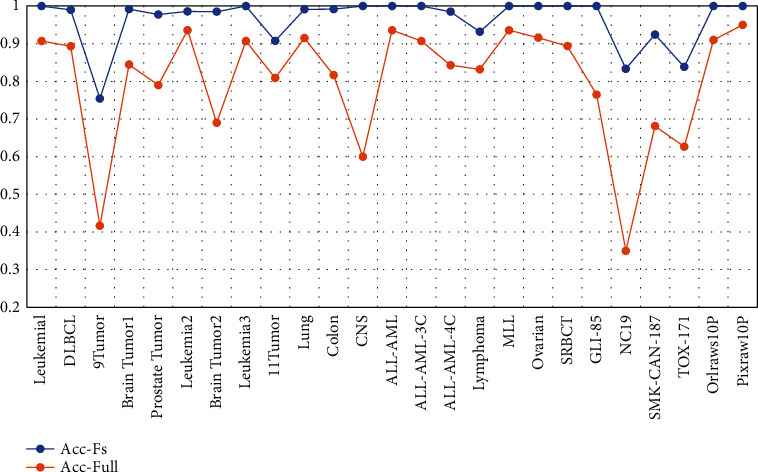
Comparison of KNN accuracy between HFIA and full.

In this section, the effectiveness of the HFIA feature selection method in improving classifier performance is verified by experiments.  Table 3 shows the quantitative comparison between the optimal feature subset (avgNfs) obtained using HFIA and the full features of the dataset.  Figure 5 depicts a comparison between the classification accuracy obtained by the KNN classifier with or without HFIA feature selection. It should be noted that the results in Table 3 and Figure 5 are the average number and average classification accuracy of the optimal feature subsets of each dataset after repeated execution 20 times.

As can be seen from Table 3, HFIA achieves a very good performance on all experimental datasets in terms of removing feature redundancy. The removal rate of feature redundancy for all datasets is above 99%. According to statistics, in all datasets participating in the experiment, the number of features of the optimal feature subset screened by HFIA is within 0.34% of the total number of features. Among them, *TOX-171* has the largest proportion of the average number of features in the optimal feature subset, with a ratio of 0.34%. *GLI-85* has the smallest proportion of the number of features in the average optimal feature subset, and its ratio is only 0.00898%.

From the perspective of improving the classification performance of the classification algorithm, in all datasets, the HFIA method improves the classifier performance by 5%–48.333%. On 52% of the datasets, HFIA improves classification performance by more than 10%. On 28% of the datasets, it improves classification performance by more than 20%. On 12% of the datasets, it improves classification performance by more than 30%. The datasets with the highest classification performance improvement are *NCI9* and *CNS*. On these datasets, the performance of the classifier is improved by more than 40%.

From the comparative analysis of the above two aspects, it can be concluded that HFIA achieves better classification accuracy than the entire feature space with a very small number of features. This fully demonstrates the effectiveness of HFIA in eliminating redundant features.

### 5.2. Comparative Analysis

In order to verify the advanced performance of the proposed algorithm, this paper compares and analyses HFIA with several feature selection algorithms reported in other literature. These feature selection methods include 5 classical feature selection algorithms and 14 hybrid feature selection methods reported in the latest literature. These comparative analyses include the following three aspects. They are the number of features and classification accuracy of the obtained optimal feature subset and the computational cost paid by the algorithm, respectively. In all tabular data, the best result for each criterion is identified in bold. It should be noted that the experimental data of the comparison algorithm are all from the corresponding literature, and our algorithm adopts the same settings as the comparative literature.

### 5.3. Comparison with Classical Feature Selection Methods

This paper conducts comparative experiments with 5 classical feature selection methods on 10 benchmark datasets. The five methods are as follows: CFS (statistical-based) [47], FCBF (information theoretical-based) [48], ReliefF (similarity-based) [49], SBMLR (sparsity-based) [50], and SPEC (graph theory-based) [51]. The experimental results are shown in Tables 4–6.  Table 4 presents a comparison of the classification accuracy of the optimal feature subsets obtained by different feature selection methods.  Table 5 describes the comparison of the number of feature subsets for different algorithms to achieve optimal accuracy.  Table 6 describes the computational cost of all algorithms to achieve optimal accuracy on these datasets. The classification accuracy data in the table is the best value obtained after 20 runs on each dataset. In this comparative experiment, all the results are obtained with the same classification algorithm and experimental parameter settings. It should be noted that the experimental data of the five classical feature selection methods in the table are all from the literature [23].

In terms of improving the performance of the classifier, the following results can be obtained from the observation and comparison of the data in Table 4. In the 10 datasets participating in the experiment, the classification accuracy of HFIA on all datasets is higher than that of the other 5 classical feature selection methods. According to the statistics in Table 3, on these datasets, the classification accuracy obtained by HFIA is 4.11%–32% higher than the maximum value of the other five algorithms. On 40% of the dataset, HFIA outperforms the maximum classification accuracy obtained by other methods by more than 10%. The highest proportion of classification accuracy is *9Tumor* and *Brain Tumor2*. On these datasets, the performance gains of the classifiers are more than 30% higher than the maximum value of other methods. This fully shows that compared with these five classical feature selection methods, the HFIA method is the best in improving the performance of the classifier.

In terms of reducing redundant features, the following results can be drawn from the data in Table 5. In these 10 datasets, the optimal feature subset obtained by HFIA has a lower number of features than other methods. It is only 2.94%–23.08% of the minimum value of other methods. On 80% of the datasets, the optimal subset obtained by HFIA has less than 15% of the minimum features of other methods. On 60% of the datasets, the number of features is below 10% of the minimum of other methods. The smallest proportion of features is *DLBCL*, *Prostate Tumor*, *Leukemia3*, and *Lung*. On these datasets, the number of features of the optimal feature subset obtained by HFIA is all below 7% of the minimum value of other methods. This fully shows that the HFIA method has the best effect in eliminating redundant features compared with these five classical feature selection methods.

In terms of the computational cost of the algorithm, the following results can be drawn from the data in Table 6. Among the five classical feature selection methods involved in the experiment, the SPEC method has the lowest computational cost. It is the fastest on all datasets. This is followed by ReliefF and FCBF methods, which are close in the computational cost on 70% of the dataset and outperform the rest of the methods. Again, the SBMLR method, which outperforms the CFS method on all datasets. Undoubtedly, the CFS method is the most computationally expensive among these 5 classical feature selection methods. By comparing the data in the table, it can be concluded that the HFIA method proposed in this paper outperforms the SBMLR method on 80% of the datasets. It outperforms FCBF and ReliefF methods on 20% of the dataset. It outperforms the CFS method on all datasets. It can be concluded that the computational cost of HFIA is between SBMLR and FCBF. This fully demonstrates that HFIA is very competitive in terms of computational cost control, even compared with classical filtering-type feature selection methods known for their speed.

After summarizing the above analysis, the following conclusions can be drawn. In terms of computational cost alone, the HFIA algorithm proposed in this paper is comparable to the classical feature selection method known for its speed. Moreover, the obtained feature subset is much better than these 5 classical feature selection methods in terms of quantity and performance improvement of the classifier. To sum up, compared with the five classical feature selection methods, the HFIA method proposed in this paper can obtain a higher-quality feature subset while taking into account the computational cost.

### 5.4. Comparison with Other Hybrid Feature Selection Methods

This paper conducts comparative experiments on 25 benchmark datasets with 14 other feature selection methods for high-dimensional data reported in the latest literature. Tables 7–9 describe the comparison of experimental results between HFIA and the feature selection method mentioned in [23]. In the literature, the authors propose an evolutionary multitask-based feature selection method (PSO-EMT) and use it to solve the classification problem of high-dimensional data. It performs well in improving classification accuracy and computational cost. This paper compares this method with 4 other feature selection algorithms on 10 gene expression datasets. These datasets all have high dimensionality, the number of features varies from 5327 to 12600, and the number of samples is small. These 4 feature selection methods are PSO, CSO, AMSO, and VLPSO. In the experiment, the classification results of the cross-validation of the KNN algorithm are used as the basis for the performance evaluation of the algorithm. The experimental results are shown in Tables 7–9.  Table 7 describes the comparison of the average classification accuracy between the HFIA algorithm and the five methods after 20 repetitions on all experimental datasets.  Table 8 describes the comparison between the average numbers of optimal feature subsets obtained by different methods.  Table 9 describes the average computational cost of all algorithms on these 10 datasets.

In terms of improving the performance of the classifier, the following results are obtained by observing and comparing the data in Table 7. Among the 10 datasets participating in the experiment, the classification accuracy of HFIA is higher than that of the other 5 feature selection methods on all datasets. After statistical analysis of the data in the table, the following results are obtained. On these datasets, the classification accuracy obtained by HFIA is 4.4%–17.77% higher than the maximum value of the other five algorithms. On 80% of the datasets, HFIA outperforms the maximum classification accuracy obtained by other methods by more than 5%. On 30% of the datasets, HFIA outperforms the maximum values of other methods by more than 10%. The highest proportion of classification accuracy is *9Tumor* and *Brain Tumor2*. On these datasets, the classification accuracy obtained by HFIA is more than 17% higher than the maximum value of other methods. This fully shows that the HFIA method is the best in improving the performance of the classifier compared with these five feature selection methods.

In terms of reducing redundant features, the following results can be drawn from the data in Table 8. In all datasets participating in the experiment, the number of features of the optimal feature subset obtained by the HFIA method is less than 50% of the minimum value of other methods. It is only 3.5%–47.52% of the minimum value of other methods. On 80% of the datasets, the number of features of the optimal feature subset obtained by the HFIA method is below 14% of the minimum value of the other methods. On 70% of the dataset, it is below 10% of the minimum of other methods. The optimal feature subset with the smallest proportion of features is *Leukemia1*, *Brain Tumor2*, *Leukemia3*, and *Lung*. On these datasets, the number of features of the optimal feature subset obtained by HFIA is less than 6% of the minimum value of other methods. This fully shows that the HFIA method has the best performance in eliminating redundant features compared with these five methods.

In terms of the computational cost of the algorithm, the following results can be drawn from the data in Table 9. Among the five feature selection methods participating in the comparative experiments, the VLPSO method has the lowest computational cost. Its computational cost is lower than the other 4 methods on 70% of the datasets. This is followed by AMSO and PSO-EMT methods, which are close in the computational cost on 60% of the dataset and outperform the rest of the methods. Next is PSO, which outperforms CSO methods on all datasets. Undoubtedly, the CSO method is the most computationally expensive of them all. By comparing the data in the table, it can be concluded that the HFIA method proposed in this paper outperforms the five methods on 90% of the datasets and only 8.6%–87.84% of the minimum value of other methods. The computational cost on 80% of the datasets is below 70% of the lowest value of other methods. The computational cost on 30% of the datasets is below 40% of the lowest value of other methods. Among them, *11Tumor* and *Lung* have the lowest computational cost, which is less than 12% of the lowest value of other methods. This fully shows that, compared with these five feature selection methods, HFIA has significant advantages in controlling the computational cost.

Through the above comparative analysis, we can draw the following summary. The optimal feature subset obtained by HFIA is better than the other five methods in average classification accuracy and average number. Moreover, its computational cost on 90% of the datasets is better than these five methods. Therefore, the following conclusions can be further drawn. Compared with these five feature selection methods, the HFIA algorithm proposed in this paper has strong competitive advantages in both the quality of the optimal feature subset and the computing speed of the algorithm. This fully proves the progressiveness of the HFIA algorithm in solving the feature selection problem of high-dimensional data.

Tables 10–12 describe the comparison of experimental results between HFIA and the feature selection method mentioned in [45]. In the literature, the authors propose a hybrid feature selection method based on the binary Jaya algorithm (TOPSIS-Jaya) and use it to solve the classification problem of high-dimensional microarray data. This method performs well in improving classification accuracy and computational cost. This paper compares this method and four other advanced feature selection algorithms on 10 microarray datasets. The four feature selection methods are HSAMB [52], CFS-iBPSO [53], PSO-DT [54], and MBEGA [55], respectively. The experimental results are shown in Tables 10–12.  Table 10 describes the comparison of the average classification accuracy of the HFIA algorithm with the 5 feature selection methods on these 10 datasets.  Table 11 describes the comparison between the average numbers of optimal feature subsets obtained by these methods.  Table 12 describes the average computational cost of all algorithms on these datasets.

In terms of improving the performance of the classifier, the following results are obtained by comparing the data in Table 10. Among the five feature selection methods involved in the comparative experiments, CFS-iBPSO obtained the highest classification accuracy, outperforming the other four methods on 70% of the datasets. The second is the TOPSIS-Jaya method, which outperforms the remaining 3 methods on 70% of the datasets. Next is the HSAMB method, which outperforms the remaining 2 methods on 80% of the datasets. Finally, there are PSO-DT and MBEGA, which perform relatively similarly on all datasets. The statistical results of the proposed HFIA method on all datasets are very similar to the CFS-iBPSO method. The average classification accuracy obtained on 70% of the datasets is greater than or equal to the best value of other methods. Moreover, its performance on 60% of the datasets achieved an average classification accuracy of 100%. This fully shows that the HFIA method has strong competitiveness in improving the performance of the classifier compared with these five feature selection methods.

In terms of the control of the number of features of the optimal feature subset, the following results can be obtained by comparing the data in Table 11. In all datasets participating in the experiment, the HFIA method outperformed the other five methods on 90% of the datasets, only 13.46%–74.24% of the minimum values of the other methods. Moreover, on 80% of the datasets, the number of features of the optimal feature subset obtained by the HFIA method is below 57% of the minimum value of other methods. On 50% of the dataset, it is below 32% of the minimum of other methods. The smallest proportion of features in the optimal feature subset is *CNS*, *ALL-AML*, and *MLL*. On these datasets, the number of optimal feature subsets obtained by HFIA is less than 28% of the minimum value of other methods. This fully shows that, compared with these five feature selection methods, the HFIA method is very advantageous in eliminating redundant features.

In terms of the computational cost of the algorithm, the following results can be obtained after comparing the data in Table 12. Among the six feature selection methods participating in the comparative experiments, the HFIA method outperforms other methods on all datasets. Moreover, its computational cost is only 17.8%–80.7% of the minimum value of other methods. Its computational cost on 60% of the datasets is below 37% of the lowest value of other methods. Its computational cost on 40% of the datasets is below 30% of the lowest value of other methods. Among them, Ovarian and Lung have the lowest computational cost, which is less than 20% of the lowest value of other methods. This fully shows that, compared with these five feature selection methods, the HFIA method has the lowest computational cost.

The following conclusions can be drawn from the above analysis. Compared with the five feature selection methods involved in the experiment, the HFIA algorithm has strong advantages in improving the classifier performance and reducing redundant features and computational cost. This shows that the HFIA algorithm can obtain higher quality feature subsets with less computational cost. This also fully proves the superiority of the HFIA algorithm proposed in this paper.

Tables 13 and 14 describe the comparison of experimental results between HFIA and the feature selection method mentioned in [56].

In the literature, the authors propose an evolutionary algorithm-based filter feature selection algorithm (TAGA) and use it to solve the classification problem of high-dimensional data. This method performs well in improving classification accuracy. This paper compares this method and 4 other algorithms on 8 benchmark datasets. The four feature selection methods are mRMR-mid [57], QPFS [58], SPECCMI [59], and CGA [60], respectively. The experimental results are shown in Tables 13 and 14.  Table 13 describes the comparison of the average classification accuracy of the HFIA algorithm with the five feature selection methods.  Table 14 describes the comparison between the average numbers of optimal feature subsets obtained by these methods.

In terms of the classification accuracy of the selected optimal feature subset, the following results are obtained by comparing the data in Table 13. Among the six feature selection methods involved in the experiment, the HFIA method proposed in this paper outperforms the other five methods on 87.5% of the datasets. On these datasets, the classification accuracy obtained by HFIA is 0.03%–18.13% higher than the optimal values of the other five algorithms. Among them, the average classification accuracy obtained on 50% of the dataset is more than 3% higher than the best value of other methods. This fully shows that, compared with these five feature selection methods, the optimal feature subset obtained by HFIA has a strong competitive advantage in classification performance.

In terms of eliminating redundant features, the following results can be drawn from the comparison of the data in Table 14. In all datasets participating in the experiment, the HFIA method outperformed the other five methods on 75% of the datasets, only 28.57%–88.57% of the minimum values of the other methods, and on 62.5% of the datasets, the number of optimal feature subsets obtained by the HFIA method is below 42% of the minimum values of other methods. The smallest proportion of features in the optimal feature subset is *Lymphoma* and *Pixraw10P*. On these datasets, the optimal number of optimal feature subsets obtained by HFIA is less than 32% of the minimum value of other methods. This fully shows that, compared with these five feature selection methods, the redundancy of the optimal feature subset selected by the HFIA method is very advantageous.

The following conclusions can be drawn from the above analysis. Compared with the five feature selection methods involved in the experiment, the HFIA algorithm has strong advantages in improving the performance of the classifier and reducing redundant features. This shows that the optimal feature subset obtained by the HFIA algorithm has higher quality. This also fully proves the superiority of the HFIA algorithm proposed in this paper.

Table 15 describes the average computational cost of HFIA and the other 4 algorithms (TAGA, SFS, BE, and CGA) on these 8 datasets. After comparing the data in Table 14, the following results can be obtained. Among the five feature selection methods involved in the experiment, the HFIA method outperforms other methods on all datasets. Moreover, its computational cost is only 4.3%–23.83% of the minimum value of other methods. Its computational cost on 75% of the datasets is below 15% of the lowest value of other methods. The computational cost on 62.5% of the datasets is below 10% of the lowest value of other methods. Among them, *SMK-CAN*-187, *TOX*-171, and *Pixraw10P* have the lowest computational cost, which is less than 7.5% of the lowest value of other methods. This fully shows that, compared with these five feature selection methods, the HFIA method has the lowest computational cost.

Through the comparative analysis of the above experimental results, it can be concluded that, combined with the evaluation results of the two indicators of the quality of the optimal feature subset and the computational cost, the HFIA method has excellent competitive advantages in feature selection of high-dimensional data compared with the 14 feature selection methods reported in the latest literature.

### 5.5. Ablation Experiments

**Figure 6:**
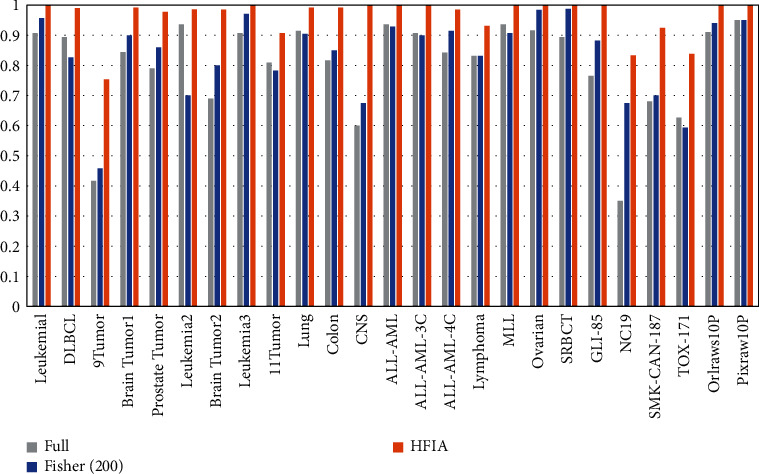
Ablation study results of the proposed method for the 25 datasets.

In order to verify the necessity of each part of the functional modules in the proposed model, ablation experiments are also performed. In this paper, ablation tests are performed on all 25 datasets participating in the experiment. For the sake of simplicity, only the classification results of the KNN algorithm are used as the analysis indicators to conduct experiments. As shown in Figure 6, the experimental results under each metric are presented. Among them, “Full” represents the method of removing all feature subset evaluation modules. “Fisher (200)” represents a module that removes the search part of the artificial immune algorithm. HFIA means the fusion of all functional modules. Through the observation and analysis of the experimental results, the following results can be obtained. From the performance of each module on the test data set, the results obtained by HFIA are significantly higher than the other two schemes and have very obvious advantages. From the performance of the KNN algorithm in classification accuracy on all datasets, the fusion method of HFIA has a better performance advantage than any other individual method. Through the above analysis, the following conclusions can be drawn. For the model proposed in this paper, the fusion scheme of HFIA is effective, which is very helpful for the performance improvement of the classifier.

## 6. Conclusion

In this paper, an efficient hybrid feature selection method (HFIA) based on an artificial immune algorithm is proposed. The algorithm combines the Fisher filter algorithm and an improved artificial immune algorithm to optimize the search process of the optimal feature subset for high-dimensional data. According to the target requirements of feature selection, the method improves the population initialization and mutation strategy of the antibody in the algorithm, as well as the population update method.

In order to verify the effectiveness of the HFIA algorithm, we conducted many experimental verifications and analyses on 25 high-dimensional datasets with features ranging from 2000 to 22283. These experimental analyses cover the following three aspects. (1) The algorithm improves the classification performance of the classifier. We compared the experimental results obtained by HFIA with the results without feature selection. These analyses include the reduction of feature redundancy and the improvement of classification accuracy. (2) Comparative analysis with other feature selection methods. We compared the experimental results with the results of 19 feature selection methods mentioned in other pieces of literature. These feature selection methods include five classical feature selection algorithms and 14 hybrid feature selection methods reported in the latest literature. These comparative analyses include classification accuracy, the number of optimal feature subsets, and the computational cost of the algorithm. (3) The structural validity analysis of the algorithm itself. To verify the necessity of each part of the functional modules in the proposed model, we conduct ablation experiments.

Based on the analysis results of the above three aspects, the following conclusions are drawn. The optimal feature subset obtained by the HFIA algorithm can improve the classification accuracy of the classifier to a great extent. Compared with the classical filtering feature selection method, the quality of the optimal feature subset obtained by the HFIA algorithm has great advantages, and its computational cost is also very competitive. Compared with the hybrid feature selection method proposed in the latest literature, the HFIA algorithm obtains the minimum number of selected feature subsets and better average classification accuracy at a lower computational cost. Therefore, it can fully illustrate the effectiveness and progressiveness of this method in solving the problem of feature selection of high-dimensional data.

In addition, it has to be said that although the HFIA algorithm has greatly improved the computational efficiency and the quality of the obtained feature subsets, there are still some problems to be solved. First, this paper uses the classification results of KNN as a criterion for evaluating the quality of candidate feature subsets. In order to obtain more accurate feature evaluation information, a fusion scheme of multiple metrics, such as rough set theory, can be considered in the evaluation of feature subsets in future research. Thirdly, because the selected optimal feature subsets are different in different classifiers, a fusion framework combining multiple feature selection algorithms and classification algorithms can be considered in order to obtain more effective results. In addition, how to obtain a better balance between classification accuracy, feature reduction rate, and computational cost is still a direction worthy of further research.

## Figures and Tables

**Algorithm 1:**
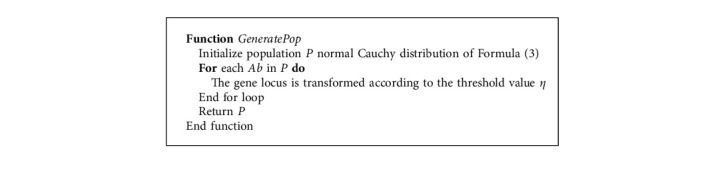
Pseudo-code of population initialization

**Algorithm 2:**
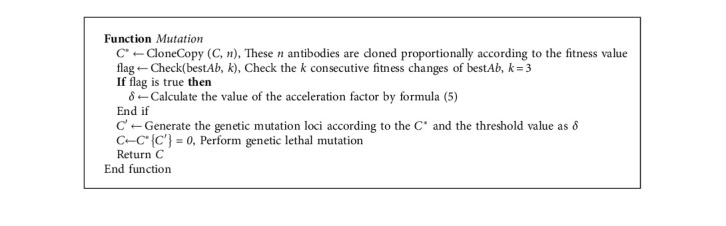
Algorithm 2 Pseudo-code of *Mutation* based on gene lethal mutation mechanism

**Algorithm 3:**
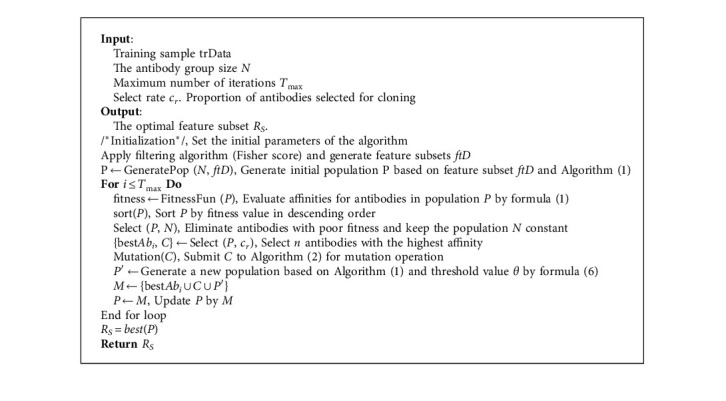
HFIA for feature selection.

**Table 1:** The description of symbols.

Symbols	Description
trData	The training sample data
*ftD*	Feature subset by the Fisher score
*P*	The set of antibodies
best*Ab*	The local optimal antibody
*R*_*S*_	Optimal feature subset returned
*N*	The population size
*n*	*n*=*c*_*r*_ × *N*, the selection pool size
fitness	The fitness of antibodies in population *P*
*δ*	The adaptive linear acceleration factor
*θ*	The linear increment factor
*c*_*r*_	Select rate
*T*_max_	The maximum number of iterations
*C*	The antibody selection set
*P′*	The antibody mutation set
*M*	Population update set
*η*	Cauchy transform threshold for initial population

**Table 2:** The summary of the experimental datasets.

Id	Dataset	Feats	Ins	Cls	Id	Dataset	Feats	Ins	Cls
1	11Tumor	12533	174	11	14	ALL-AML-3C	7129	72	3
2	9Tumor	5726	60	9	15	ALL-AML-4C	7129	72	4
3	Brain Tumor1	5920	90	5	16	Lymphoma	4026	96	9
4	Brain Tumor2	10367	50	4	17	MLL	12582	72	3
5	DLBCL	5469	77	2	18	Ovarian	15154	253	2
6	Leukemia1	5327	72	3	19	SRBCT	2308	83	4
7	Leukemia2	7129	72	4	20	GLI-85	22283	85	2
8	Lung	12600	203	5	21	NCI9	9712	60	9
9	Prostate tumor	10509	102	2	22	SMK-CAN-187	19993	187	2
10	Leukemia3	11225	72	3	23	TOX-171	5748	171	4
11	Colon	2000	62	2	24	Orlraws10P	10304	100	10
12	CNS	7129	60	2	25	Pixraw10P	10000	100	10
13	ALL-AML	7129	72	2					

*Note.* Feats, Ins, and Cls represent features, instances, and classes, respectively.

**Table 3:** Quantitative comparison between the optimal feature subset of HFIA and full features.

Id	Dataset	Features	avgNfs/std	ID	Dataset	Features	avgNfs/std
1	Leukemia1	5327	1.8/0.41	14	ALL-AML-3C	7129	1.8/0.41
2	DLBCL	5469	2.15/2.11	15	ALL-AML-4C	7129	3.6/1.9
3	9Tumor	5726	17.95/4.64	16	Lymphoma	4026	6.95/3.34
4	Brain Tumor1	5920	12.75/3.12	17	MLL	12582	1.8/0.41
5	Prostate tumor	10509	2.5/1.15	18	Ovarian	15154	1.65/0.49
6	Leukemia2	7129	7.4/5.34	19	SRBCT	2308	2.8/0.7
7	Brain Tumor2	10367	3.6/1.9	20	GLI-85	22283	2/0.67
8	Leukemia3	11225	1.95/0.6	21	NCI9	9712	15/6.19
9	11Tumor	12533	24.35/3.1	22	SMK-CAN-187	19993	6.2/2.94
10	Lung	12600	7.35/4.85	23	TOX-171	5748	19.6/7.87
11	Colon	2000	1.7/0.82	24	Orlraws10P	10304	3.7/0.95
12	CNS	7129	1/0	25	Pixraw10P	10000	2/0
13	ALL-AML	7129	1.1/0.32				

**Table 4:** The comparison of the best classification accuracy.

Dataset	CFS	FCBF	ReliefF	SBMLR	SPEC	HFIA
Leukemia1	95.89	91.61	94.46	93.04	94.29	**100**
DLBCL	91.96	93.21	92.82	92.75	74.11	**100**
9Tumor	56.67	41.67	61.67	48.33	38.33	**91.667**
Brain Tumor1	86.67	80	85.78	82.22	84.44	**100**
Prostate tumor	94.09	91.12	92.09	95.09	85.36	**100**
Leukemia2	88.57	85.89	91.29	84.64	90	**100**
Brain Tumor2	68	64	68	64	48	**100**
Leukemia3	94.01	93.14	94.29	93.04	84.29	**100**
11Tumor	83.91	82.94	84.91	70.13	83.3	**97.059**
Lung	93.31	92.06	90.17	92.62	81.29	**100**

**Table 5:** Comparison of the number of optimal feature subsets obtained by different methods.

Dataset	CFS	FCBF	ReliefF	SBMLR	SPEC	HFIA
Leukemia1	97	49	603	14	2489	**1**
DLBCL	88	66	343	34	2371	**1**
9Tumor	47	32	544	26	1049	**6**
Brain Tumor1	142	106	771	27	5388	**4**
Prostate tumor	59	49	433	32	405	**1**
Leukemia2	119	71	645	18	3544	**2**
Brain Tumor2	117	75	1171	21	4420	**2**
Leukemia3	138	80	978	17	4917	**1**
11Tumor	379	394	1114	15	6158	**13**
Lung	550	453	1440	30	2378	**2**

**Table 6:** Computational cost comparison of different feature selection methods.

Dataset	CFS	FCBF	ReliefF	SBMLR	SPEC	HFIA
Leukemia1	741.57	1.49	1.3	0.92	0.19	4.8145
DLBCL	685.15	1.52	1.37	2.01	0.34	4.194
9Tumor	652.7	1.53	1.11	7.14	0.41	5.4977
Brain Tumor1	1248.17	2.69	2.04	9.2	0.66	5.8385
Prostate tumor	572.82	3.57	2.21	9.84	0.83	4.9926
Leukemia2	810.32	1.68	1.63	10.79	0.99	5.255
Brain Tumor2	765.5	2.84	1.54	11.5	1.16	5.3947
Leukemia3	677.51	4.04	2.84	13.46	1.4	5.341
11Tumor	4681.4	25.06	15.54	13.87	2.28	7.0041
Lung	10029	37.95	16.69	28.13	3.11	6.2198

**Table 7:** Comparison of classification accuracy of optimal feature subsets obtained by different methods.

Dataset	PSO	CSO	AMSO	VLPSO	PSO-EMT	HFIA
Leukemia1	80.60/2.55	90.79/2.88	94.01/1.58	93.31/2.34	91.11/2.79	**100/0**
DLBCL	83.67/1.52	94.60/3.26	94.10/1.95	86.51/2.88	93.76/2.80	**99/2.44**
9Tumor	42.72/1.42	59.78/3.55	50.11/3.61	54.94/4.80	58.00/4.02	**75.42/7.39**
Brain Tumor1	73.73/2.21	80.41/3.93	72.67/3.79	71.19/3.52	87.37/1.50	**99.17/2.04**
Prostate tumor	84.50/1.64	79.95/3.18	89.58/1.35	88.74/2.23	89.65/1.82	**97.75/2.55**
Leukemia2	78.61/2.02	80.83/2.28	87.52/2.00	85.82/2.96	90.07/2.47	**98.57/3.01**
Brain Tumor2	61.99/2.91	80.73/5.62	74.96/3.48	66.78/4.10	72.27/4.09	**98.5/3.66**
Leukemia3	89.83/1.00	91.49/3.84	94.45/1.04	91.56/1.67	94.51/1.50	**100/0**
11Tumor	71.81/1.75	83.50/1.70	83.10/1.31	80.92/2.39	86.15/1.45	**90.74/4.19**
Lung	78.77/1.53	88.94/1.75	89.97/1.80	89.55/1.68	91.09/0.94	**99.13/1.6**

**Table 8:** Comparison of the number of optimal feature subsets obtained by different methods.

Dataset	PSO	CSO	AMSO	VLPSO	PSO-EMT	HFIA
Leukemia1	2615.5	170.12	51.49	54.7	198.4	**1.8/0.41**
DLBCL	2681	30.08	50.56	48.14	83.55	**2.15/2.11**
9Tumor	2811.9	220.34	52.16	47.05	263.09	**17.95/4.637**
Brain Tumor1	2917.2	207.61	93.54	26.83	351.21	**12.75/3.117**
Prostate tumor	2926.6	207.98	44.36	35.97	149.86	**2.5/1.147**
Leukemia2	3513.8	389.4	71.54	53.39	224.44	**7.4/5.336**
Brain Tumor2	5117.2	90.43	62.08	81.46	499.69	**3.6/1.903**
Leukemia3	5535.7	88.64	57.19	35.23	268.08	**1.95/0.605**
11Tumor	6205	589.36	319	249.3	541.45	**24.35/3.104**
Lung	6234.7	230.41	193.47	176	617.61	**7.35/4.848**

**Table 9:** Computational cost comparison of different feature selection methods.

Dataset	PSO	CSO	AMSO	VLPSO	PSO-EMT	HFIA
Leukemia1	41.2	247.29	6.8	6.09	9.28	**5.3494/0.252**
DLBCL	47.59	389.67	8.34	7.18	7.02	**4.7939/0.34**
9Tumor	39.18	370.4	**5.52**	5.65	8.09	6.4389/0.396
Brain Tumor1	66.65	457.24	11.65	9.55	15.43	**6.4799/0.303**
Prostate tumor	78.77	410.83	14.31	11.05	16.88	**5.5481/0.441**
Leukemia2	66.09	445.99	9.66	8.96	12.19	**6.0048/0.485**
Brain Tumor2	80.5	945.7	12.06	11.76	11.51	**5.7796/0.334**
Leukemia3	120.64	1837.91	15.64	15.94	14.72	**5.7177/0.223**
11Tumor	418.54	6278.54	91.22	67.41	106.53	**7.5514/0.651**
Lung	574.17	5419.71	255.32	78	134.59	**6.7417/0.313**

**Table 10:** Comparison of classification accuracy of optimal feature subsets obtained by different methods.

Dataset	TOPSIS-Jaya	HSAMB	CFS-iBPSO	PSO-DT	MBEGA	HFIA
Colon	97.76	90.27	94.89	90.32	86.66	**99.17/0.026**
CNS	96.22	84.17	95.84	58.33	72.21	**100/0**
ALL-AML	**100**	99.34	**100**	95.83	95.89	**100/0**
ALL-AML-3C	**100**	99.18	**100**	95.83	96.64	**100/0**
ALL-AML-4C	**99.72**	96.79	97.63	94.44	91.39	98.5/0.037
Lung	94.24	–	**100**	**100**	98.96	99.13/0.016
Lymphoma	98.33	99.99	**100**	98.5	97.68	93.16/0.027
MLL	99.62	99.55	**100**	94.04	94.33	**100/0**
Ovarian	99.52	99.81	**100**	97.23	99.71	**100/0**
SRBCT	**100**	99.57	**100**	92.49	99.23	**100/0**

**Table 11:** Comparison of the number of optimal feature subsets obtained by different methods.

Dataset	TOPSIS-Jaya	HSAMB	CFS-iBPSO	PSO-DT	MBEGA	HFIA
Colon	18.9	4.16	4.2	643.3	24.5	**1.7/0.823**
CNS	8.7	7.43	10.5	1486	20.5	**1/0**
ALL-AML	16.1	5	4.3	1468	15.8	**1.1/0.316**
ALL-AML-3C	6.6	5.84	6	1294.1	20.1	**1.8/0.41**
ALL-AML-4C	19.5	6.37	20.7	1845	26.2	**3.6/1.903**
Lung	9.9	—	10.6	1657	14.1	**7.35/4.848**
Lymphoma	15.2	**3.75**	24	1346	34.3	6.95/3.342
MLL	12.9	6.6	30.8	4847	32.1	**1.8/0.41**
Ovarian	18.5	5.73	3.3	3594.2	9	**1.65/0.489**
SRBCT	15.8	8.9	34.1	874	60.7	**2.8/0.696**

**Table 12:** Computational cost comparison of different feature selection methods.

Dataset	TOPSIS-Jaya	HSAMB	CFS-iBPSO	MBEGA	HFIA
Colon	12.71	142	39.27	70.6	**3.7616/0.563**
CNS	14.79	101	78.43	81.1	**8.9231/0.51**
ALL-AML	15.07	102	141.48	112.3	**3.8569/0.234**
ALL-AML-3C	16.4	233	204.15	176.6	**12.3797/0.789**
ALL-AML-4C	16.7	141	321.33	234.3	**5.7796/0.334**
Lung	35.03	—	311.22	1041.7	**6.7417/0.313**
Lymphoma	17.42	92	366.24	142.6	**14.0521/0.523**
MLL	18.33	152	245.71	182.1	**12.8591/1.083**
Ovarian	68.49	3000	92.9	2689.5	**12.2007/0.796**
SRBCT	16.38	188	302.81	246.2	**6.0331/0.259**

**Table 13:** Comparison of classification accuracy of optimal feature subsets obtained by different methods.

Algorithm	CLN	GLI	NCI	SMK	TOX	LYM	ORP	PIW
TAGA	94.03/0.8	98.8/0.0	79.5/0.4	75.2/0.5	77.7/0.8	94.3/0.5	99.8/0.4	97.0/0.0
mRMR-mid	98.4	95.3	65	68.4	72.5	**97.9**	97	96
QPFS	91.9	94.1	83.3	74.3	72.5	**97.9**	98	97
SPECCMI	93.5	96.5	80	71.1	77.2	93.8	94	95
CGA	95.2/1.3	95.9/0.6	78.8/2.4	72.7/1.5	74.3/2.6	94.1/1.0	98.5/1.0	97.2/0.4
HFIA	**99.17/2.64**	**100/0**	**83.33/5.56**	**92.43/3.32**	**83.82/3.73**	93.16/2.69	**100/0**	**100/0**

*Note.* CLN, GLI, NCI, SMK, TOX, LYM, ORP, and PIW in the table represent the datasets Colon, GLI-85, NCI9, SMK-CAN-187, TOX-171, Lymphoma, Orlraws10P, and Pixraw10P, respectively.

**Table 14:** Comparison of the number of optimal feature subsets obtained by different methods.

Algorithm	CLN	GLI	NCI	SMK	TOX	LYM	ORP	PIW
TAGA	10.6/8.1	14.8/4.3	40.5/3.5	13.1/4.4	23.6/6.5	20.3/3.7	13.4/4.7	8.1/0.7
mRMR-mid	**1**	5	43	**7**	28	22	20	7
QPFS	3	12	39	17	**15**	23	9	20
SPECCMI	32	16	38	13	24	36	21	11
CGA	7.9/3.9	16.2/7.0	39.0/5.8	8.8/6.3	28.8/3.9	30.3/7.0	13.6/3.6	10.2/5.1
HFIA	1.7/0.82	**2/0.67**	**15/11.19**	**6.2/2.94**	19.6/17.87	**6.95/3.34**	**3.7/0.95**	**2/0**

**Table 15:** Computational cost comparison of different feature selection methods.

Algorithm	CLN	GLI	NCI	SMK	TOX	LYM	ORP	PIW
TAGA	123	122	127	139	132	117	129	148
SFS	34	48	33	140	123	59	62	63
BE	183	223	162	649	587	294	288	301
CGA	158	181	168	183	174	161	199	183
HFIA	**3.76/0.56**	**4.46/0.2**	**5.79/0.06**	**9.73/0.88**	**5.29/0.19**	**14.05/0.52**	**5.69/0.27**	**4.66/0.06**

## Data Availability

The datasets were taken from UCI Repository; Arizona State University; https://github.com/primekangkang/Genedata (microarray datasets); and https://ckzixf.github.io/dataset.html.
